# Holistic Type Extension for Classical Logic via Toffoli Quantum Gate

**DOI:** 10.3390/e21070636

**Published:** 2019-06-27

**Authors:** Hector Freytes, Roberto Giuntini, Giuseppe Sergioli

**Affiliations:** 1 Dipartimento di Filosofia, University of Cagliari, Via Is Mirrionis 1, 09123 Cagliari, Italy; 2 Centro Linceo Interdisciplinare “B. Segre”, 00165 Roma, Italy

**Keywords:** quantum computational logic, fuzzy logic, quantum Toffoli gate, 02.10.Ab, 02.10.De

## Abstract

A holistic extension of classical propositional logic is introduced via Toffoli quantum gate. This extension is based on the framework of the so-called “quantum computation with mixed states”, where also irreversible transformations are taken into account. Formal aspects of this new logical system are detailed: in particular, the concepts of tautology and contradiction are investigated in this extension. These concepts turn out to receive substantial changes due to the non-separability of some quantum states; as an example, Werner states emerge as particular cases of “holistic” contradiction.

## 1. Introduction

In recent years, an increasing interest in logical systems related to quantum mechanics has arisen. Most of these systems are not strictly related to the standard quantum logic, but they are motivated by concrete problems related to quantum information and quantum computation [1,2,3,4,5,6,7].

The notion of quantum computation first appeared in the 1980s by Richard Feynman. One of the central issues he posed was the difficulty to efficiently simulate the evolution of a quantum system on a classical computer. He pointed out the computational benefits that arise by employing quantum systems in place of classical ones. With this aim, he proposed a new kind of computer: a *quantum computer* [8]. It was not conceived as a Turing machine, but as a different kind of machine able to simulate any quantum system, including the physical world. Quantum computing can simulate all computations that can be performed by classical systems. However, one of the main advantages of quantum computation and quantum algorithms is that they can speed up computations.

In classical computation, information is encoded by a sequence of bits. A bit is viewed as a kind of physical object that can assume one of two distinct classical states, represented by the binary numbers 0 or 1. Bits are manipulated via an ensemble of logical gates like *NOT*, *OR*, *AND*, etc, arranged in circuits and providing the output of a calculation.

Standard quantum computing is based on quantum systems described by finite dimensional Hilbert spaces, starting from $C^{2}$, which is the two-dimensional space where any qubit lives. A qubit—the unit of information in quantum computation—is represented by a unit vector in $C^{2}$, while *n*-qubits (where *n* is a positive integer) are represented by unit vectors in $C^{2^{n}}$. Similarly to the classical case, we can introduce and study the behaviour of a certain number of quantum gates acting on *n*-qubits. These quantum gates are mathematically modelled by unitary operators applying on pure states of an appropriate Hilbert space and thus they only represent reversible processes. However, for many reasons, this restriction to unitary operators is undue. In fact, a quantum system is rarely in a pure state. This may be caused, for example, by the incomplete efficiency in the preparation procedure and also by manipulations on the system as measurements over pure states; in both cases, we are faced with *statistical mixtures*. Such restriction induced the formulation of more general models for quantum computation, where density operators and quantum operations are employed in place of pure states and unitary operators. This approach to quantum computing, where not only reversible transformations are taken into account, is called *quantum computation with mixed states*.

In this powerful model, combinational structures associated with a set of quantum gates induce new forms of quantum logical systems [7] that play a similar role to Boolean algebras with respect to digital circuits. We focus our attention on the combinational structure of quantum circuits built from a particular quantum gate: the *Toffoli quantum gate*.

The study of the combinational logic underlying the Toffoli quantum gate is interesting for several reasons. One of these is related to the universality of quantum gates. In particular, the Toffoli gate alone is universal for classical computation and, equipped with the Hadamard gate, is approximately universal for quantum computation [9], i.e., a finite composition of the Toffoli and Hadamard gate allows representing the behavior of any other quantum gate.

However, another reason that makes the logic of the Toffoli gate interesting is its connection with fuzzy logic. Indeed, from a probabilistic point of view, the Toffoli gate behaves as the conjunction of *Product logic* [10]. This logical system is related to the so-called *fuzzy logic of continuous t-norms* introduced in the second part of the 1990s [11].

Focusing on the Toffoli quantum gate, the aim of this paper is to study an extension of classical logic that arises from the holistic nature of bipartite quantum systems.

The paper is structured as follows: the first two sections provide all the necessary ingredients to make the article self-contained. More precisely, in Section 2, we introduce some basic notion concerning non-separability and bipartite quantum systems. In Section 3, we briefly describe the mathematical model related to quantum computation with mixed states. In Section 4, we introduce the general logical framework associated with quantum circuits. This new form of quantum logic is compared to the standard quantum logic based on the closed subspaces of the Hilbert space, also-called *Hilbert lattices*.

Section 5 is devoted to studying the fuzzy extension arisen from Toffoli gate. This extension will be defined by means of two particular instances of Toffoli gate: AND and NOT. In Section 6, a holistic type extension for classical logic is investigated. This extension is motivated by the application of AND gate on non-separable states. In Section 7, we study the notion of contradiction in the holistic extension of classical logic and in Section 8 Werner states are introduced as particular cases of these contradictions. Finally, in Section 9, some arguments and possible open discussions are briefly introduced as conclusive remarks.

## 2. Bipartite Quantum Systems

In quantum mechanics, a compound system is represented as a tensor product of Hilbert spaces, each of them representing the individual parts of the system. Unlike classical physics, standard quantum mechanics systematically violate the above separability principle. This difference arises from the tensor product structure related to Hilbert spaces and from the superposition principle [12,13,14]. More precisely, if $\rho_{1}$ and $\rho_{2}$ are two density operators in the Hilbert spaces $H_{1}$ and $H_{2}$, respectively, the state of the compound system is represented by $\rho =\rho_{1}⊗\rho_{2}$ in $H_{1}⊗H_{2}$. However, not all density operators on $H_{1}⊗H_{2}$ are expressible in this form. The property of non-factorizability of quantum states is given by the fact that the direct sum of $H_{1}$ and $H_{2}$ is a proper subset of $H_{1}⊗H_{2}$. In what follows, we introduce some notation that turns out to be very useful to describe our *holistic extension* of the classical logic based on the Toffoli quantum gate.

Let us remind readers that any density operator ρ can be expressed as $\rho =\frac{1}{2}\left(I+s_{1}\sigma_{1}+s_{2}\sigma_{2}+s_{3}\sigma_{3}\right)$, where *I* is the identity matrix, $\sigma_{1},\sigma_{2},\sigma_{3}$ are Pauli matrices and $s_{1},s_{2}$ and $s_{3}$ are three real numbers such as $s_{1}^{2}+s_{2}^{2}+s_{3}^{2}\leq 1$. The triple $\left(s_{1},s_{2},s_{3}\right)$ represents a point of the Bloch sphere uniquely associated with the density operator ρ. Similarly, it can be obtained for any *n*-dimensional Hilbert space by the generalized Pauli-matrices.

**Definition** **1.** *Let H be a n-dimensional Hilbert space and $\{|\psi_{1}\rangle ,\ldots ,|\psi_{n}\rangle \}$ be the canonical orthonormal basis of H. Let us consider k, j be two natural numbers such that: 1≤k<j≤n. Then, the generalized Pauli-matrices are defined as follows:*

$${}^{\left(n\right)}\sigma_{1}^{\left[k,j\right]}=|\psi_{j}\rangle \langle \psi_{k}\left|+\right|\psi_{k}\rangle \langle \psi_{j}|$$

$${}^{\left(n\right)}\sigma_{2}^{\left[k,j\right]}=i(|\psi_{j}\rangle \langle \psi_{k}\left|-\right|\psi_{k}\rangle \langle \psi_{j}|)$$

*and for* 1≤k≤n−1

$${}^{\left(n\right)}\sigma_{3}^{\left[k\right]}=\sqrt{\frac{2}{k(k+1)}}(|\psi_{1}\rangle \langle \psi_{1}\left|+\cdots +\right|\psi_{k}\rangle \langle \psi_{k}\left|-k\right|\psi_{k+1}\rangle \langle \psi_{k+1}|).$$

If $H=C^{2}$, one immediately obtains: ${}^{\left(2\right)}\sigma_{1}^{\left[1,2\right]}=\sigma_{1}$, ${}^{\left(2\right)}\sigma_{2}^{\left[1,2\right]}=\sigma_{2}$ and ${}^{\left(2\right)}\sigma_{3}^{\left[1\right]}=\sigma_{3}.$

Let ρ be a density operator of the *n*-dimensional Hilbert space H. For each *j* satisfying $1\leq j\leq n^{2}-1$, let us consider

$$s_{j}\left(\rho \right)=tr\left(\rho \sigma_{j}\right).$$

The sequence $\langle s_{1}\left(\rho \right)\ldots s_{n^{2}-1}\left(\rho \right)\rangle$ is called the *generalized Bloch vector* associated with ρ, in view of the following well-known result [15]: let ρ be a density operator of the *n*-dimensional Hilbert space H and let $\sigma_{j}\in P_{n}$. Then, the density operator ρ can be represented as:

(1) $$\rho =\frac{1}{n}I^{\left(n\right)}+\frac{1}{2}\sum_{j=1}^{n^{2}-1}s_{j}\left(\rho \right)\sigma_{j},$$

where $I^{\left(n\right)}$ is the n×n identity matrix.

By using generalized Pauli matrices, it will be possible to formally describe a notion of holism for bipartite states. In fact, by the Schlienz–Mahler decomposition [15], we can show how any quantum bipartite state can be expressed as a sum of a factorizable state plus another quantity that represents a kind of holistic component.

Let us consider the Hilbert space $H=H_{a}⊗H_{b}$. For each density operator ρ on H, we shall denote by $\rho_{a}$ the partial trace of ρ with respect to the subsystem $H_{b}$ (i.e., $\rho_{a}=tr_{H_{b}}\left(\rho \right)$) and, similarly, by $\rho_{b}$ the partial trace of ρ with respect to the subsystem $H_{a}$ (i.e., $\rho_{b}=tr_{H_{a}}\left(\rho \right)$). For the next sections, let us recall the following result:

Let ρ be a density operator in the *n*-dimensional Hilbert space $H=H_{a}⊗H_{b}$ such that $dim(H_{a})=m$ and $dim(H_{b})=k$. By dividing ρ in m×m blocks $B_{i,j}$, each of them is a *k*-square matrix, then:

(2) $$\begin{array}{lll} \rho_{a} & = & tr_{H_{b}}\left(\rho \right)=\left[\begin{array}{cccc} trB_{1,1} & trB_{1,2} & \ldots & trB_{1,m}\\ trB_{2,1} & trB_{2,2} & \ldots & trB_{2,m}\\ \vdots & \vdots & \vdots & \vdots \\ trB_{m,1} & trB_{m,2} & \ldots & trB_{m,m} \end{array}\right] \end{array}$$

(3) $$\begin{array}{lll} \rho_{b} & = & tr_{H_{a}}\left(\rho \right)=\sum_{i=1}^{m}B_{i,i}. \end{array}$$

**Definition** **2.** *Let ρ be a density operator in the Hilbert space $H_{m}⊗H_{k}$, where $dim(H_{m})=m$ and $dim(H_{k})=k$. Then, ρ is (m,k)-factorizable iff $\rho =\rho_{m}⊗\rho_{k}$ where $\rho_{m}$ is a density operator in $H_{m}$ and $\rho_{k}$ is a density operator in $H_{k}$*.

Note that, if ρ is (m,k)-factorizable as $\rho =\rho_{m}⊗\rho_{k}$, this factorization is unique and $\rho_{m}$ and $\rho_{k}$ correspond to the reduced states of ρ on $H_{m}$ and $H_{k}$, respectively [16].

Let us suppose that $H=H_{a}⊗H_{b}$ where $dim(H_{a})=m$ and $dim(H_{b})=k$. Consider the generalized Pauli matrices $\sigma_{1}^{a},\ldots ,\sigma_{m^{2}-1}^{a}$ and $\sigma_{1}^{b},\ldots ,\sigma_{k^{2}-1}^{b}$ arising from $H_{a}$ and $H_{b}$, respectively. By defining the coefficients:

$$M_{j,l}\left(\rho \right)=tr\left(\rho \left[\sigma_{j}^{a}⊗\sigma_{l}^{b}\right]\right)-tr\left(\rho \left[\sigma_{j}^{a}⊗I^{\left(k\right)}\right]\right)tr\left(\rho \left[I^{\left(m\right)}⊗\sigma_{l}^{b}\right]\right)$$

and by considering the matrix M(ρ) defined as

$$M\left(\rho \right)=\frac{1}{4}\sum_{j=1}^{m^{2}-1}\sum_{l=1}^{k^{2}-1}M_{j,l}\left(\rho \right)\left(\sigma_{j}^{a}⊗\sigma_{l}^{b}\right)$$

then M(ρ) represents the “additional component” of ρ when ρ is not a factorized state. Thus, if ρ is a density operator in $H=H_{a}⊗H_{b}$, then

(4) $$\rho =\rho_{a}⊗\rho_{b}+M\left(\rho \right).$$

The above result provides a mathematical representation of the instance of holism mentioned at the beginning of the section. Indeed, a state ρ in $H_{a}⊗H_{b}$ does not only depend on its reduced states $\rho_{a}$ and $\rho_{b}$, but the summand M(ρ) is also involved. We notice that M(ρ) is not a density operator and then it does not represent a physical state. We refer to M(ρ) as the *holistic component* of ρ.

## 3. Quantum Computation with Mixed States

As anticipated in the Introduction, we now provide some basic notions of quantum computing. In quantum computation, information is elaborated and processed by means of quantum systems. A *quantum bit* or *qubit*, the fundamental concept of quantum computation, is a pure state in the Hilbert space $C^{2}$. The standard orthonormal basis {|0〉,|1〉} of $C^{2}$ is called *logical basis*. They are related to the fact that the logical truth is represented by |1〉 and the falsity by |0〉. Therefore, a pure state |ψ〉 in $C^{2}$ can be written as $|\psi \rangle =c_{0}|0\rangle +c_{1}|1\rangle$, where $c_{0}$ and $c_{1}$ are complex numbers such that $|c_{0}{|}^{2}+{\left|c_{1}\right|}^{2}=1$. Recalling the Born rule, any qubit $|\psi \rangle =c_{0}|0\rangle +c_{1}|1\rangle$ may be regarded as a piece of information, where the number $|c_{0}{|}^{2}$ corresponds to the probability-value of the information described by the basic state |0〉, while $|c_{1}{|}^{2}$ corresponds to the probability-value of the information described by the basic state |1〉. The two basis-elements |0〉 and |1〉 are usually taken as the encoding of the classical bit-values 0 and 1, respectively. In this way, the qubit probability value we are interested in is $p(|\psi \rangle )=|c_{1}{|}^{2}$, which is related to the basis vector associated with truth.

Quantum states considered in quantum computation live in the tensor product ${⊗}^{n}C^{2}=C^{2}⊗C^{2}⊗\ldots ⊗C^{2}$ (*n* times) that is a $2^{n}$-dimensional complex space. A special basis, called the $2^{n}$-*computational basis*, is chosen for ${⊗}^{n}C^{2}$. In other words, it consists of the $2^{n}$ orthogonal states |ι〉, $0\leq \iota \leq 2^{n}$ where ι is in binary representation and |ι〉 is a tensor product of states (Kronecker product) $\left|\iota \rangle =\right|\iota_{1}\rangle ⊗|\iota_{2}\rangle ⊗\ldots ⊗|\iota_{n}\rangle$, whit $\iota_{j}\in \left\{0,1\right\}$. Then, a pure state $|\psi \rangle \in {⊗}^{n}C^{2}$ can be written as $|\psi \rangle =\sum_{\iota =1}^{2^{n}}c_{\iota }|\iota \rangle$, with $\sum_{\iota =1}^{2^{n}}{\left|c_{\iota }\right|}^{2}=1$.

In the usual representation of quantum computational processes, a quantum circuit is identified with an appropriate composition of *quantum gates*, mathematically represented by *unitary operators* acting on pure states of a convenient (*n*-fold tensor product) Hilbert space ${⊗}^{n}C^{2}$ [17]. In other words, the standard model for quantum computation is mathematically based on “*qubits-unitary operators*”.

As we said in the Introduction, in general, a quantum system is not in a pure state. Moreover, there are interesting processes that cannot be encoded by unitary evolutions. For example, the measurement at the end of the computation is a non-unitary operation, and the final state is a probability distribution over pure states, i.e., a mixed state.

In this way, several authors [5,6,7,18,19] have considered a general model for quantum computing, where pure states are changed with mixed states. In what follows, we provide a brief description of this powerful model for quantum computers based on mixed states, which is better suited to our development.

As a particular case, we may associate to each vector of the logical basis of $C^{2}$ two density operators $P_{0}=\left|0\rangle \langle 0\right|$ and $P_{1}=\left|1\rangle \langle 1\right|$ that represent, in this framework, the falsity-property and the truth-property, respectively. Let us consider the operator $P_{1}^{\left(n\right)}={⊗}^{n-1}I⊗P_{1}$ on ${⊗}^{n}C^{2}$. By applying the Born rule, we shall consider the probability of a density operator ρ as follows:

(5) $$p\left(\rho \right)=Tr\left(P_{1}^{\left(n\right)}\rho \right).$$

Note that, in the particular case in which ρ=|ψ〉〈ψ|, where $|\psi \rangle =c_{0}|0\rangle +c_{1}|1\rangle$, we obtain that $p\left(\rho \right)=|c_{1}{|}^{2}$. Thus, this probability value associated with ρ is the generalization of the probability value considered for qubits.

A *quantum operation* [20] is a linear operator $E:L\left(H_{1}\right)\to L\left(H_{2}\right)$ where $L(H_{i})$ is the space of linear operators in the complex Hilbert space $H_{i}$ (i=1,2), representable as $E\left(\rho \right)=\sum_{i}A_{i}\rho A_{i}^{†}$ where $A_{i}$ are operators satisfying $\sum_{i}A_{i}^{†}A_{i}=I$ (Kraus representation). It can be seen that a quantum operation maps density operators into density operators. Each unitary operator *U* can be described as has a quantum operation $O_{U},$ where, for any density operator ρ, $O_{U}\left(\rho \right)=U\rho U^{†}$. Thus, quantum operations generalize unitary operators. It provides a powerful model for quantum computation in which irreversible processes can also be considered. This model founded on density operators and quantum operations is known as “*quantum computation with mixed states*” ([7,18]).

## 4. Standard Quantum Logic vs. Quantum Computational Logic

The holistic extensions for classical logic in quantum computing, announced as the main goal of this paper, is fully supported in the formalism of quantum computation with mixed states. This naturally suggests a kind of quantum logical system related to quantum computation that allows us to achieve the extension mentioned above. As expected, this logical system will be substantially different than standard Birkhoff–von Neumann quantum logic [21]. In this section, we summarize the differences between these two logical systems.

According to von Neumann’s axiomatization, quantum events are mathematically realized by projectors of a Hilbert space H. Hence, any experimental proposition concerning a quantum system corresponds to a projector in a convenient Hilbert space. Closed subspaces of H are in one-to-one correspondence with the class of all projectors of H and they form an algebra called *Hilbert lattice* (denoted by L(H)). In any Hilbert lattice, the meet operation ∧ corresponds to the set theoretical intersection between subspaces and the join operation ∨ corresponds to the smallest closed subspace of H containing the set theoretical union of subspaces. The ordering relation associated with the lattice L(H) is the inclusion of subspaces. Note that L(H) is a bounded lattice where H is the *maximum*, denoted by 1, while 0 denotes the *minimum*, i.e., the subspace containing only the origin. This lattice equipped with the relation of orthogonal complement ${}^{⊥}$ can be described as an *ortholattice* [22]. Then, the propositional structure that defines the standard quantum logic proposed by Birkhoff and von Neumann is given by the ortholattice $\langle L(H),\vee ,\wedge {,}^{⊥},1,0\rangle$. Let us notice that, unlike classical logic, in this structure, the distributive law fails. However, L(H) satisfies a kind of weak distributivity. In case of a finite-dimensional Hilbert space H, the ortholattice L(H) is modular, i.e., satisfies the following condition known as *modular law*: x≤y⟹x∨(y∧z)=y∧(x∨z). In the case of an infinite-dimensional Hilbert space, the modular law is not satisfied. In 1937, Husimi [23] showed that a weaker law, the so-called *orthomodular law* ($x\leq y⟹x\vee (x^{⊥}\wedge y)=y$), is satisfied in the ortholattice L(H).

Quantum computational logic can be considered as a different kind of quantum logic. It arises from the combinational structure associated with a set of quantum gates, mathematically represented by quantum operations. Let us remember that the mathematical support for quantum computation is given by finite dimensional Hilbert spaces of the form ${⊗}^{n}C^{2}$. While the standard quantum logic associated with a system represented by ${⊗}^{n}C^{2}$ is given by the ortholattice $L({⊗}^{n}C^{2})$, on the contrary, possible quantum computational logic systems are defined taking into account algebraic properties of quantum operations acting over density operators on ${⊗}^{n}C^{2}$. Although it is clear that these logical systems are not Boolean, their notion of logical consequence is inspired by the following problem, usually treated in classical computation and more precisely in digital techniques. If *T* is a combinational circuit, we want to know whether a determinate input state of *T*, represented by a string of bits 0 and 1, forces a determinate output state of *T* given by a bit that could be either 0 or 1. As a general rule, this problem can be solved through effective procedures based on classical logic.

Then, one may naturally extend this problem by considering circuits made from assemblies of quantum gates. In this way, the input and the output of quantum circuits are labeled by density operators and possible notions of logical consequence are defined by relations between the input and the output of circuits. Several families of quantum computational logics arise from these extensions [5,6,24]. These families of logics have a common semantics based on probability-values introduced in Equation (5). More precisely, a language for a quantum computational logic is a propositional language $L_{F}\left(X\right)$ where *X* is a non-empty set of variables and F is a set of connectives. Propositional variables are interpreted in a set D of density operators and, for each connective f∈F, *f* is naturally interpreted as a quantum operation $U_{f}$ closed on D. An interpretation of $L_{F}\left(X\right)$ in D is any function $e:L_{F}\left(X\right)\to D$ such that, for any f∈F having arity *k*, $e\left(f\left(x_{1},\ldots ,x_{k}\right)\right)=U_{f}\left(e\left(x_{1}\right),\ldots ,e\left(x_{k}\right)\right)$. To define a relation of semantic consequence ⊧ based on the probability assignment, it is necessary to introduce the notion of valuations. In fact, *valuations* are functions over the unitary real interval $v:L_{F}\left(X\right)\to \left[0,1\right]$ such that *f* can be factorized in the following way:
(6)
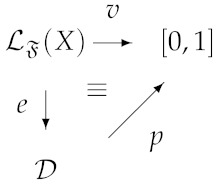


Since an interpretation always determines a valuation, for each interpretation *e*, we denote by $e_{p}$ the valuation related to *e*. The abstract notion of semantical consequence ⊧ related to D is given by:

α⊧φiffR[v(α),v(φ)],

where $R\subseteq {\left[0,1\right]}^{2}$ is a reflexive and transitive relation. Note that the natural extension of classical logical consequence can be formulated as follows:

(7) $$\alpha ⊧\varphi \;iff\;e_{p}\left(\alpha \right)=1⟹e_{p}\left(\varphi \right)=1.$$

More precisely, in this case, $R[e_{p}\left(\alpha \right),e_{p}\left(\varphi \right)]$ is defined by $e_{p}\left(\alpha \right)=1⟹e_{p}\left(\varphi \right)=1$. These kinds of logical systems can be framed as generalizations of probabilistic logic. *Probabilistic logic* is the concept that Adams [25] introduced for the logical investigation on the transmission of probability values thorough valid inferences. In our context, the notion of probabilistic logial system can be generalized by considering non-Kolmogorovian probability models [26] as it happens in the case of quantum computational logics, which is semantically based on the Born rule.

## 5. A Fuzzy Extension for Classical Logic in Quantum Computation

As introduced in the previous section, the probabilistic semantic for a language $L_{F}\left(X\right)$ associated with quantum computational logic assumes its truth value in the continuous [0,1]. This suggests a strong relation between quantum computational logic and fuzzy logic. Since we are interested in an extension of classical logic in a quantum computational framework, it is quite natural for above-mentioned logical systems to require the following condition: once a language $L_{F}\left(X\right)$ is fixed, the elements of the set F have to be interpreted as quantum operations that are able to fully describe, from the truth-functionally point of view, classical logic. In other words, the set of connectives F, restricted to the classical truth values {0,1}, is functionally complete (we say that a set of classical connectives is *functionally complete* if it is sufficient to express every truth-function) with respect to propositional classical logic. In this section, we shall consider a logical system equipped with only one connective, semantically interpreted as the well-known Toffoli quantum gate.

### 5.1. Classical Functional Completeness and the Extension via Toffoli Gate

Functionally completeness, besides being an important logical property, turns out to be crucial also for technological applications. A paradigmatic case is represented by the digital techniques where logical gates can be represented by propositional connectives and circuits by propositional formulas. For technical reasons (standardization of integrated circuits, energy optimization), sometimes it is necessary to build circuits by using a restricted set of logical gates. We focus our attention on the set 〈¬,∧〉, which is functionally complete for classical logic. Thus, by induction, a logical system 〈¬,∧〉 can represent all truth-functions of classical logic. However, the set 〈¬,∧〉 could not be functionally complete for some extension of classical logic. The rest of this subsection is devoted to investigating a natural extension of 〈¬,∧〉 to quantum computational logic with mixed states.

First of all, the classical negation is extended in the following way:

**Definition** **3.** *For each density operator ρ in ${⊗}^{m}C^{2}$, the negation ${\mathrm{NOT}}^{\left(2^{m}\right)}\left(\rho \right)$ is defined as follows:*

$${\mathrm{NOT}}^{\left(2^{m}\right)}\left(\rho \right)=\left(I^{\left(2^{m-1}\right)}⊗NOT\right)\;\rho \;\left(I^{\left(2^{m-1}\right)}⊗NOT\right),$$

*where* $NOT=\left[\begin{array}{cc} 0 & 1\\ 1 & 0 \end{array}\right].$

In [27], it is proved that

(8) $$p({\mathrm{NOT}}^{\left(2^{m}\right)}\left(\rho \right))=1-p\left(\rho \right).$$

An extension of the classical conjunction can be implemented via the *Toffoli gate*. It was introduced by Tommaso Toffoli [28] and it is represented by the ternary classical connective $T\left(x,y,z\right)=\left(x,y,xy\hat{+}z\right),$ where $\hat{+}$ is the sum modulo 2. When z=0, T(x,y,0) reproduces the classical conjunction. Toffoli gate is naturally extended to qubits in the following way.

For any natural numbers m,k≥1 and for any vectors of the standard basis $\left|x\rangle =\right|x_{1}\ldots x_{m}\rangle \in {⊗}^{m}C^{2}$, $\left|y\rangle =\right|y_{1}\ldots y_{k}\rangle \in {⊗}^{k}C^{2}$ and $|z\rangle \in C^{2}$, the Toffoli gate $T^{\left(m,k,1\right)}$ on ${⊗}^{m+k+1}C^{2}$ is defined as follows:

$$T^{\left(m,k,1\right)}(|x\rangle ⊗|y\rangle ⊗|z\rangle )=\left|x\rangle ⊗|y\rangle ⊗\right|x_{m}y_{k}\hat{+}z\rangle .$$

By ([29] Proposition 3.1), for any choice of m,k≥1, $T^{\left(m,k,1\right)}$ is a unitary operator whose matrix representation is given by

(9) $$\begin{array}{lll} T^{\left(m,k,1\right)} & = & I^{\left(2^{m+k+1}\right)}+P_{1}^{\left(2^{m}\right)}⊗P_{1}^{\left(2^{k}\right)}⊗\left(Not-I\right) \end{array}$$

(10) $$\begin{array}{lll} & = & I^{\left(2^{m-1}\right)}⊗\left[\begin{array}{cc} I^{\left(2^{k+1}\right)} & 0\\ 0 & I^{\left(2^{k-1}\right)}⊗Xor \end{array}\right], \end{array}$$

where $Xor=\left[\begin{array}{cccc} 1 & 0 & 0 & 0\\ 0 & 1 & 0 & 0\\ 0 & 0 & 0 & 1\\ 0 & 0 & 1 & 0 \end{array}\right]$.

$T^{\left(m,k,1\right)}$ allows us to extend the classical conjunction as follows.

**Definition** **4.** *Let $\rho^{m}$ be a density operator in ${⊗}^{m}C^{2}$ and $\rho^{k}$ be a density operator in ${⊗}^{k}C^{2}.$ We define:*

$${\mathrm{AND}}^{\left(m,k\right)}\left(\rho^{m}⊗\rho^{k}\right)=T^{\left(m,k,1\right)}\left(\rho^{m}⊗\rho^{k}⊗P_{0}\right)T^{\left(m,k,1\right)}.$$

In [27], it is proved that

(11) $$p\left({\mathrm{AND}}^{\left(m,k\right)}\left(\rho^{m}⊗\rho^{k}\right)\right)=p\left(\rho^{m}\right)p\left(\rho^{k}\right).$$

Let us consider the set $D_{n}$ of all density operators on ${⊗}^{n}C^{2}$. It is very important to remark that ${\mathrm{AND}}^{\left(m,k\right)}$ can be seen as a binary operator of the form

(12) $${\mathrm{AND}}^{\left(m,k\right)}:D_{m}\times D_{k}\to D_{m+k+1}.$$

In order to define a quantum computational logical system in the sense of Section 3 and based on $\left\{{\mathrm{AND}}^{\left(-,-\right)},{\mathrm{NOT}}^{\left(2^{-}\right)}\right\}$, we consider the set $D={⋃}_{n}D_{n}$ and we introduce the binary connective AND and the unary connective NOT in D as

$\mathrm{AND}\left(\rho ,\sigma \right)={\mathrm{AND}}^{\left(m,k\right)}\left(\rho ⊗\sigma \right)$ iff, $\rho \in D_{m}$ and $\sigma \in D_{k},$
$\mathrm{NOT}\left(\rho \right)={\mathrm{NOT}}^{\left(2^{m}\right)}\left(\rho \right)$ iff $\rho \in D_{m}$.

Note that AND and NOT are closed operations in D. Thus, these operations define a quantum computational logical system in the sense of Section 3 that we shall denote as ${\mathrm{QC}}_{AN}$. By Equations (8) and (11), it is immediate to see that

(13) p(NOT(ρ))=1−p(ρ),p(AND(ρ,σ))=p(ρ)p(σ).

From a probabilist point of view, ${\mathrm{NOT}}^{\left(2^{m}\right)}$ gate can be described as an instance of Toffoli gate. In fact, by ([29] Theorem 3.1), for each density operator ρ in ${⊗}^{m}C^{2}$, we can easily see that

$$p\left({\mathrm{NOT}}^{\left(2^{m}\right)}\left(\rho \right)\right)=p\left(T^{\left(m,k,1\right)}\left(\rho ⊗P_{1}^{\left(k\right)}⊗P_{1}\right)T^{\left(m,k,1\right)}\right).$$

Thus, AND and NOT can be considered as two particular instances of Toffoli gate. Consequently, ${\mathrm{QC}}_{AN}$ can be seen as a logic construction arising from Toffoli gate only.

In the case where p(ρ) and p(σ) are 1 or 0, these quantum gates behave as the classical negation and conjunction, respectively. In this way, ${\mathrm{QC}}_{AN}$ provides an extension of the classical propositional logic.

It is possible to characterize the subset of D for which the set of connectives {NOT,AND} classically behaves. In fact: let $\rho \in D_{n}$ and suppose that the diagonal of ρ is given by $diag\left(\rho \right)=\left\{r_{1,1},r_{2,2}\ldots r_{2^{n},2^{n}}\right\}$. Note that p(ρ)∈{0,1} iff $\sum_{i=1}^{2^{n-1}}r_{2i,2i}\in \left\{0,1\right\}$. If we define the set

$$D_{n}^{class}=\left\{\rho ={\left(r_{i,j}\right)}_{1\leq i,j\leq 2^{n}}\in D_{n}:\sum_{i=1}^{2^{n-1}}r_{2i,2i}\in \left\{0,1\right\}\right\},$$

then

(14) $$D^{class}=\underset{n}{⋃}D_{n}^{class}$$

is the subset of D in which {NOT,AND} classically behaves.

### 5.2. ${\mathrm{QC}}_{AN}$ and the Connection with the Fuzzy Logic

In the general case, ${\mathrm{QC}}_{AN}$ is strongly related to the *Basic fuzzy logic* introduced by Hájek at the end of the 1990s [11]. This kind of fuzzy logic is conceived as a theory of the approximate reasoning based on many-valued logic systems. Basic fuzzy logic is the logic associated with *continuous t-norms* i.e., continuous, commutative, associative, and monotone binary operations on [0,1] with 1 as the neutral element. These operations are taken as possible truth-functions of conjunctions in these systems. Each continuous *t*-norm determines a semantics of fuzzy propositional logic. For example, the *Łukasiewcz t-norm*$x{⊙}_{Ł}y=\max\left\{0,x+y-1\right\}$ defines the conjunction of the Łukasiewcz infinite many valued logic, where $\neg_{Ł}x=1-x$ is the negation in this logic. The *product t-norm* $x{⊙}_{p}y=x\cdot y$ defines the conjunction of the Product logic [10] and the Gödel *t*-norm $x{⊙}_{p}y=\min\left\{x,y\right\}$ defines the conjunction of the linear Heyting logic. In this subsection, we investigate the formal relation between ${\mathrm{QC}}_{AN}$ and the fuzzy logic system based on the product *t*-norm.

Since p(NOT(ρ))=1−p(ρ), we can identify NOT with the Łukasiewicz negation and since p(AND(ρ,σ))=p(ρ)p(σ), AND can be identified with the product *t*-norm. Thus, from a strictly semantic point of view, we can establish the following identification:

(15) $$\left\{\mathrm{NOT},\mathrm{AND}\right\}\overset{\approx }{\mathrm{semantic}}\left\{\neg_{Ł},{⊙}_{p}\right\}.$$

We remark that connectives $\left\{\neg_{Ł},{⊙}_{p}\right\}$ define a multiplicative fragment of the fuzzy logical system known as *product many valued logic or PMV-logic*, studied in [30,31].

This semantic connection between two logical systems is even deeper and it is formally rooted in the equivalence relation on D given by

(16) ρ≈σiffp(ρ)=p(σ).

It is not very hard to see that the quotient set $D{/}_{\approx }$ can be identified to the real interval [0,1] and ≈ is a congruence with respect to {NOT,AND}. Thus, both operations naturally induce two operations over the equivalence classes in $D{/}_{\approx }$ given by ${\mathrm{NOT}}_{\approx }\left(\left[\rho \right]\right)=\left[\mathrm{NOT}\left(\rho \right)\right]$ and ${\mathrm{AND}}_{\approx }\left(\left[\rho \right],\left[\sigma \right]\right)=\left[\mathrm{AND}\left(\rho ,\sigma \right)\right]$. Then, the algebraic structures $\langle D_{\approx },{\mathrm{NOT}}_{\approx },{\mathrm{AND}}_{\approx }\rangle$ and $\langle \left[0,1\right],\neg_{Ł},{⊙}_{p}\rangle$ coincide and they induce the same algebraic semantic for both logical systems. As a consequence, the natural {NOT,AND}-homomorphism $\pi :D\to D{/}_{\approx }=\left[0,1\right]$ is identifiable with the assignment of probability in ${\mathrm{QC}}_{AN}$. In this way, ${\mathrm{QC}}_{AN}$ is semantically related to basic fuzzy logic providing a fuzzy extension for the propositional classical logic in quantum computation with mixed states.

### 5.3. Extending Classical Contradictions and Tautologies in ${\mathrm{QC}}_{AN}$

In classical logic, concepts of contradiction and tautology can be syntactically represented in terms of {¬,∧}. Contradictions are those formulas equivalent to p∧¬p and tautologies are those formulas equivalent to ¬(p∧¬p). From these facts, the formula p∧¬p is sometimes referred to as *syntactic contradiction* and ¬(p∧¬p) (more precisely, the equivalent form p∨¬p) is referred to as *syntactic tautology*. In this work, we accord with this terminology.

In ${\mathrm{QC}}_{AN}$, a syntactic representation for contradictions and tautologies is lost. This fact can be explained taking into account that real numbers do not contain zero divisors. Then, there is not an algebraic expression built from $\left\{\neg_{Ł},{⊙}_{p}\right\}$ that produces the constant functions 1 or 0. Hence, the semantic identification given in Equation (15) does not exist a formula in the language of {AND,NOT} that produces a contradiction or a tautology in ${\mathrm{QC}}_{AN}$. However, the ${\mathrm{QC}}_{AN}$-extensions of the syntactic contradiction and the syntactic tautology have interesting properties. The ${\mathrm{QC}}_{AN}$ are:

$p\wedge \neg p\;\overset{{\mathrm{QC}}_{AN}}{⟶}\;\mathrm{AND}\left(\rho ,\mathrm{NOT}\rho \right)$     [syntactic contradiction],
$\neg \left(p\wedge \neg p\right)\;\overset{{\mathrm{QC}}_{AN}}{⟶}\;\mathrm{NOT}\left(\mathrm{AND}\left(\rho ,\mathrm{NOT}\rho \right)\right)$ [syntactic tautology].

Since NOT is an involution on D, the ${\mathrm{QC}}_{AN}$-extension of the syntactic contradiction and ${\mathrm{QC}}_{AN}$-extension of the syntactic tautology are dual concepts. Thus, for the sake of simplicity, we can focus our attention on the notion of contradiction only. By Equation (15), we can see that:

(17) $$p\left(\mathrm{AND}\left(\rho ,\mathrm{NOT}\rho \right)\right)=p\left(\rho \right)\left(1-p\left(\rho \right)\right)\leq \frac{1}{4}.$$

Thus, p(AND(ρ,NOTρ))=0 iff p(ρ)∈{0,1}. In other words, the fuzzy extension of the classic syntactic contradiction AND(−,NOT(−)) has a classical behaviour over the set $D^{class}$ only.

## 6. A Holistic Type Extension for Classical Logic

Quantum computational logic with mixed states can also provide an interesting holistic type extension for the classical propositional logic. This extension arises when non factorizable states are considered as inputs in the Toffoli quantum gate. We will also note that the fuzzy system $\left\{\neg_{Ł},{⊙}_{P}\right\}$ plays an important role for describing the mentioned holistic extension.

The formal language in which classical logic and most of the logical systems are expressed are regulated by strict syntax rules. The basic idea at the origin of these languages is the fact that each proposition or formula can be built by means of a recursive procedure from a distinguished set of propositions, which are called atomics propositions. In this way, complex propositions are recursively obtained from atomic propositions assembled by connectives. For each connective a natural number, the *arity* is assigned. The arity defines the number of propositions that the connectives assemble. When an algebraic semantic for these logical systems is considered, an *n*-ary connective is interpreted as an algebraic operation having *n* arguments. Thus, the arity is an invariant property associated with a connective. All of these ideas were already taken into account in ${\mathrm{QC}}_{AN}$, where separability conditions of the states were considered. More precisely, ${\mathrm{AND}}^{\left(m,k\right)}$ is viewed as a 2-ary connective in the ideal case in which a factorizable state of the form $\rho_{m}⊗\rho_{k}$ is considered as input.

In general, of course, this is not the case. Quantum systems continually interact with environment, building up correlations. For a more realistic approach, we can assume that the input of the ${\mathrm{AND}}^{\left(m,k\right)}$ can be also a non-factorizable mixed state ρ in ${⊗}^{m+k}C^{2}$ taking into account its holistic type representation given in Equation (4), i.e.,

$$\rho =\rho_{m}⊗\rho_{k}+M\left(\rho \right),$$

where $\rho_{m}$ and $\rho_{k}$ are the reduced states of ρ in ${⊗}^{m}C^{2}$ and ${⊗}^{k}C^{2}$, respectively.

Unlikely, with respect to Equation (12), when non factorized states are taken into account, ${\mathrm{AND}}^{\left(m,k\right)}$ behaves as a unary operator of the form ${\mathrm{AND}}^{\left(m,k\right)}:D_{m+k}\to D_{m+k+1}$. This behavior of ${\mathrm{AND}}^{\left(m,k\right)}$ motivates a holistic type extension of classical conjunction. The following definition formally introduces an operator that describes the unary behavior of ${\mathrm{AND}}^{\left(m,k\right)}$.

**Definition** **5.** *For any density operator $\rho \in {⊗}^{m+k}C^{2}$, we define:*

$${\mathrm{AND}}_{Hol}^{\left(m,k\right)}\left(\rho \right)=T^{\left(m,k,1\right)}\left(\rho ⊗P_{0}\right)T^{\left(m,k,1\right)}.$$

For the sake of the simplicity, we use the following notation: if ρ is a density operator in ${⊗}^{m+k}C^{2}$, then $T_{p}^{\left(m,k,1\right)}\left(\rho \right)$ denotes the matrix

$$T_{p}^{\left(m,k,1\right)}\left(\rho \right)=P_{1}^{2^{m+k+1}}\left(T^{\left(m,k,1\right)}\left(M\left(\rho \right)⊗P_{0}\right)T^{\left(m,k,1\right)}\right).$$

Then, by Equations (4) and (11), it follows that, if ρ is a density operator in ${⊗}^{m+k}C^{2}$ and $\rho_{m}$, $\rho_{k}$ are the reduced states of ρ in ${⊗}^{m}C^{2}$ and ${⊗}^{k}C^{2}$, respectively, then:

(18) $${\mathrm{AND}}_{Hol}^{\left(m,k\right)}\left(\rho \right)={\mathrm{AND}}^{\left(m,k\right)}\left(\rho_{m}⊗\rho_{k}\right)+T^{\left(m,k,1\right)}\left(M\left(\rho \right)⊗P_{0}\right)T^{\left(m,k,1\right)}$$

and the probability of the holistic conjunction will assume the form:

(19) $$p\left({\mathrm{AND}}_{Hol}^{\left(m,k\right)}\left(\rho \right)\right)=p\left(\rho_{m}\right)p\left(\rho_{k}\right)+tr\left(T_{p}^{\left(m,k,1\right)}\left(\rho \right)\right).$$

Furthermore, in the special case where $\rho =\rho_{m}⊗\rho_{k}$, Equation (18) clearly collapses in:

(20) $${\mathrm{AND}}_{Hol}^{\left(m,k\right)}\left(\rho \right)={\mathrm{AND}}^{\left(m,k\right)}\left(\rho_{m}⊗\rho_{k}\right).$$

The above result shows that ${\mathrm{AND}}^{\left(m,k\right)}$ is implicitly acting in ${\mathrm{AND}}_{Hol}^{\left(m,k\right)}\left(\rho \right)$ over the reduce states of ρ.

In what follows, we provide a simple way to estimate $p({\mathrm{AND}}_{Hol}^{\left(m,k\right)}\left(\rho \right))$, $p(\rho_{m})$, $p(\rho_{k})$ and $tr(T_{p}^{\left(m,k,1\right)}\left(M\left(\rho \right)\right))$. We first introduce the following technical definition.

**Definition** **6.** *Let $\rho ={\left(r_{i,j}\right)}_{1\leq i,j\leq 2^{m+k}}$ be a density operator in ${⊗}^{m+k}C^{2}$ divided in $2^{m}\times 2^{m}$ blocks $T_{i,j}$ such that each of them is a $2^{k}$-square matrix:*

$$\rho =\left(\begin{array}{cccc} T_{1,1} & T_{1,2} & \ldots & T_{1,2^{m}}\\ T_{2,1} & T_{2,2} & \ldots & T_{2,2^{m}}\\ \vdots & \vdots & \vdots & \vdots \\ T_{2^{m},1} & T_{2^{m},2} & \ldots & T_{2^{m},2^{m}} \end{array}\right).$$

Then, the (m,k)-*Toffoli blocks of ρ* are the diagonal blocks ${\left(T_{i}=T_{i,i}\right)}_{1\leq i\leq 2^{m}}$ of ρ. For the sake of formal semplicity, we also introduce the following quantities:

$\beta^{m,k}\left(\rho \right)=\sum_{j=1}^{2^{m}-1}\sum_{i=0}^{2^{k-1}-1}r_{\left(2i+1\right)+j2^{k}}$ that is the sum of the odd diagonal elements of the even (m,k)-Toffoli blocks $T_{2i}$ of ρ,
$\gamma^{m,k}\left(\rho \right)=\sum_{j=0}^{2^{m}-2}\sum_{i=1}^{2^{k-1}}r_{2i+j2^{k}}$ that is the sum of the even diagonal elements of the odd (m,k)-Toffoli blocks $T_{2i+1}$ of ρ,
$\delta^{m,k}\left(\rho \right)=\sum_{j=1}^{2^{m}-1}\sum_{i=1}^{2^{k-1}}r_{2i+j2^{k}}$ that is the sum of the odd diagonal elements of the odd (m,k)-Toffoli blocks $T_{2i+1}$ of ρ.

By ([29] Proposition 4.3) for each density operator ρ in ${⊗}^{m+k}C^{2}$ where m,k≥1 and with $r_{i}$*i*-th diagonal element of ρ, then:

(21) $$p\left({\mathrm{AND}}_{Hol}^{\left(m,k\right)}\left(\rho \right)\right)=\sum_{j=1}^{2^{m-1}}\sum_{i=1}^{2^{k-1}}r_{\left(2j-1\right)2^{k}+2i}.$$

More precisely, $p({\mathrm{AND}}_{Hol}^{\left(m,k\right)}\left(\rho \right))$ is the sum of the even diagonal elements of the even (m,k)-Toffoli blocks $T_{2i}$ of ρ.

Equation (21) is a useful tool that allows us to evaluate in very simple way all the terms involved in Equation (19), as the next theorem provides (for more technical details, see ([29] Proposition 4.4.))

**Theorem** **1.** *Let ρ be a density operator in ${⊗}^{m+k}C^{2}$. Let $\rho_{m}$ and $\rho_{k}$ be the reduced states of ρ on ${⊗}^{m}C^{2}$ and ${⊗}^{k}C^{2}$, respectively. Then,*

1. *$1=p\left({\mathrm{AND}}_{Hol}^{\left(m,k\right)}\left(\rho \right)\right)+\beta^{m,k}\left(\rho \right)+\gamma^{m,k}\left(\rho \right)+\delta^{m,k}\left(\rho \right)$,*
2. *$p\left(\rho_{m}\right)=p\left({\mathrm{AND}}_{Hol}^{\left(m,k\right)}\left(\rho \right)\right)+\beta^{m,k}\left(\rho \right)$,*
3. *$p\left(\rho_{k}\right)=p\left({\mathrm{AND}}_{Hol}^{\left(m,k\right)}\left(\rho \right)\right)+\gamma^{m,k}\left(\rho \right)$,*
4. *$tr\left(T_{p}^{\left(m,k,1\right)}\left(\rho \right)\right)=p\left({\mathrm{AND}}_{Hol}^{\left(m,k\right)}\left(\rho \right)\right)\delta^{m,k}\left(\rho \right)-\beta^{m,k}\left(\rho \right)\gamma^{m,k}\left(\rho \right)$.*

Interestingly enough, Theorem 1 allows us to obtain some boundary estimation on the quantities involved in Equation (19).

By Theorem 1-(2 and 3) is immediate to see that

(22) $$p\left(AND_{Hol}^{\left(m,n\right)}\left(\rho \right)\right)\leq p\left(\rho_{m}\right),p\left(\rho_{k}\right).$$

Furthermore, the incidence of the holistic component M(ρ) on the probability of $p(AND_{Hol}^{\left(m,n\right)}\left(\rho \right))$ lives in the bounded interval:

(23) $$-\frac{1}{4}\leq tr\left(T_{p}^{\left(m,k,1\right)}\left(\rho \right)\right)\leq \frac{1}{4}.$$

To show this, we have to consider the following maximum/minimum problem

$$\left\{\begin{array}{c} tr\left(T_{p}^{\left(m,k,1\right)}\left(\rho \right)\right)=\delta^{m,k}{\left(\rho \right)}^{2}-\beta^{m,k}\left(\rho \right)\gamma^{m,k}\left(\rho \right),\\ 2\delta^{m,k}\left(\rho \right)+\beta^{m,k}\left(\rho \right)+\gamma^{m,k}\left(\rho \right)=1. \end{array}\right.$$

Note that $\max tr(T_{p}^{\left(m,k,1\right)}\left(\rho \right))$ is given when $\beta^{m,k}\left(\rho \right)+\gamma^{m,k}\left(\rho \right)=0$. Thus, $\max\left\{tr\left(T_{p}^{\left(m,k,1\right)}\left(\rho \right)\right)\right\}=\delta^{m,k}{\left(\rho \right)}^{2}=\frac{1}{4}$. While $\min\{tr\left(T_{p}^{\left(m,k,1\right)}\left(\rho \right)\right)\}$ is given under the condition $\beta^{m,k}\left(\rho \right)+\gamma^{m,k}\left(\rho \right)=1$. In this way, $\min\left\{tr\left(T_{p}^{\left(m,k,1\right)}\left(\rho \right)\right)\right\}-\max\left\{\beta^{m,k}\left(\rho \right)\left(1-\beta^{m,k}\left(\rho \right)\right)\right\}=-\frac{1}{4}$.

Finally, in the special case where $p(AND_{Hol}^{\left(m,k\right)}\left(\rho \right))=1$ the holistic component of ρ has not any probability incidence, i.e., $tr(T_{p}^{\left(m,k,1\right)}\left(\rho \right))=0$. In this case, $p\left(\rho^{m}\right)=p\left(\rho^{k}\right)=1$. In fact, suppose that $p(AND_{Hol}^{\left(m,k\right)}\left(\rho \right))=1$; then, by Theorem 1-(1 and 2), $p\left(\rho^{m}\right)=p\left(\rho^{k}\right)=1$ and $\beta^{m,k}\left(\rho \right)=\gamma^{m,k}\left(\rho \right)=0$. Thus, $1=p\left({\mathrm{AND}}_{Hol}^{\left(m,k\right)}\left(\rho \right)\right)+\delta^{m,n}\left(\rho \right)+\beta^{m,k}\left(\rho \right)+\gamma^{m,k}\left(\rho \right)=1+\delta^{m,n}\left(\rho \right)+0+0$ and then $\delta^{m,n}\left(\rho \right)=0$. Hence, by Theorem 1-3, $tr(T_{p}^{\left(m,k,1\right)}\left(\rho \right))=0$.

To define an holistic extension of the classical conjunction starting from ${\mathrm{AND}}_{Hol}^{\left(m,k\right)}$, we have to deal with the following situation: if ρ is a density operator on ${⊗}^{n}C^{2}$ where $n=m+k=m^{\prime }+k^{\prime }$ and $m\neq m^{\prime },k\neq k^{\prime }$, then we generally have that

$${\mathrm{AND}}_{Hol}^{\left(m,k\right)}\left(\rho \right)\neq {\mathrm{AND}}_{Hol}^{\left(m^{\prime },k^{\prime }\right)}\left(\rho \right).$$

In other words, a logical connective based on ${\mathrm{AND}}_{Hol}^{\left(-,-\right)}$ also requires a precise information about the holistic representation of the argument in the sense of Equation (4). For this, we introduce the following notions: $\rho_{\langle m,k\rangle }$ indicates that ρ is a density operator in ${⊗}^{m+k}C^{2}$ where the holistic representation $\rho =\rho_{m}⊗\rho_{k}+M\left(\rho \right)$ is chosen. We also define the set $D_{Hol}$ as:

$$D_{Hol}=\left\{\rho_{\langle m,k\rangle }:m,k\in N\right\}.$$

If we consider the relation in $D_{Hol}$ given by

(24) $$\rho_{\langle m,k\rangle }\approx_{H}\rho_{\langle m^{\prime },k^{\prime }\rangle }\;\mathrm{iff}\;m+k=m^{\prime }+k^{\prime },$$

then $\approx_{H}$ is an equivalence and $D_{Hol}{/}_{\approx_{H}}=D$.

We also note that, if ρ is a density operator on ${⊗}^{m+k}C^{2}$, Proposition 9 suggests a privileged (holistic) interpretation of the codomain for ${\mathrm{AND}}_{Hol}^{\left(m,k\right)}\left(\rho \right)$. In fact:

$$\begin{array}{lll} {\mathrm{AND}}_{Hol}^{\left(m,k\right)}\left(\rho \right) & = & T^{\left(m,k,1\right)}\left(\rho ⊗P_{0}\right)T^{\left(m,k,1\right)}\\ & = & \left(I^{\left(2^{m+k+1}\right)}+P_{1}^{\left(2^{m}\right)}⊗P_{1}^{\left(2^{k}\right)}⊗\left(Not-I\right)\right)\left(\rho ⊗P_{0}\right)(I^{\left(2^{m+k+1}\right)}+\\ & & P_{1}^{\left(2^{m}\right)}⊗P_{1}^{\left(2^{k}\right)}⊗\left(Not-I\right))\\ & = & \rho ⊗P_{0}+M\left({\mathrm{AND}}_{Hol}^{\left(m,k\right)}\left(\rho \right)\right), \end{array}$$

where $M\left({\mathrm{AND}}_{Hol}^{\left(m,k\right)}\left(\rho \right)\right)=P_{1}^{\left(2^{m}\right)}⊗P_{1}^{\left(2^{k}\right)}⊗\left(Not-I\right))\left(\rho ⊗P_{0}\right)\left(P_{1}^{\left(2^{m}\right)}⊗P_{1}^{\left(2^{k}\right)}⊗\left(Not-I\right)\right)$. This suggests to consider ${\left({\mathrm{AND}}_{Hol}^{\left(m,k\right)}\left(\rho \right)\right)}_{\langle m+k,1\rangle }$ as a natural holistic representation for ${\mathrm{AND}}_{Hol}^{\left(m,k\right)}\left(\rho \right)$. Thus, we define the holistic extension of the classical conjunction as follows:

$${\mathrm{AND}}_{Hol}\left(\rho_{\langle m,k\rangle }\right)={\left({\mathrm{AND}}_{Hol}^{\left(m,k\right)}\left(\rho \right)\right)}_{\langle m+k,1\rangle }$$

In this way, ${\mathrm{AND}}_{Hol}$ defines a unary connective in $D_{Hol}$. Note that Equation (18) provides a deep relation between the connectives ${\mathrm{AND}}_{Hol}$ and AND. In fact, for $\rho_{\langle m,k\rangle }=\rho_{m}⊗\rho_{k}+M\left(\rho \right),$ we have that

$$\begin{array}{lll} {\mathrm{AND}}_{Hol}\left(\rho_{\langle m,k\rangle }\right) & = & {\mathrm{AND}}^{\left(m,k\right)}\left(\rho_{m}⊗\rho_{k}\right)+T^{\left(m,k,1\right)}\left(M\left(\rho \right)⊗P_{0}\right)T^{\left(m,k,1\right)}\\ & = & \mathrm{AND}\left(\rho_{m}⊗\rho_{k}\right)+T^{\left(m,k,1\right)}\left(M\left(\rho \right)⊗P_{0}\right)T^{\left(m,k,1\right)}. \end{array}$$

The connective NOT, formally defined on D, has a natural extension to $D_{Hol}$. Taking into account the equivalence $\approx_{H}$ in $D_{Hol}$, introduced in Equation (24), for each $\rho_{\langle m,k\rangle }\in D_{Hol},$ we can define $\mathrm{NOT}\left(\rho_{\langle m,k\rangle }\right)=\mathrm{NOT}\left({\left[\rho_{\langle m,k\rangle }\right]}_{\approx_{H}}\right)$ where the equivalence class ${\left[\rho_{\langle m,k\rangle }\right]}_{\approx_{H}}$ is identified to a density operator on D. In this way, $\approx_{H}$ becomes a congruence with respect to NOT, and NOT is well defined on $D_{Hol}$.

The pair ${\mathrm{AND}}_{Hol}$, NOT defines a holistic type extension for classical logic in the framework of quantum computation with mixed states. We denote this logical system as ${\mathrm{QC}}_{AN}^{Hol}$. We want to remark two peculiarities about the system ${\mathrm{QC}}_{AN}^{Hol}$. First: while classical logic needs at least one binary connective to describe any possible truth-function, ${\mathrm{QC}}_{AN}^{Hol}$ can describe any possible classical truth-function by involving two unary connectives. Second: since ${\mathrm{QC}}_{AN}^{Hol}$ is described by unary connectives, the notion of classical syntactic contradiction—that had a natural extension in ${\mathrm{QC}}_{AN}$—seems to not have an extension in ${\mathrm{QC}}_{AN}^{Hol}$. The next section is devoted to this topic.

## 7. Syntactic Contradiction in ${\mathrm{QC}}_{AN}^{Hol}$

${\mathrm{QC}}_{AN}^{Hol}$ is a logical system having unary connectives only. This fact does not allow us to extend, in a natural way, the syntactic representation of the classical contradiction given by p∧¬p. However, it is possible to characterize a sub class of $D_{Hol}$ that preserves the notion of syntactic contradiction when ${\mathrm{AND}}_{Hol}$ takes arguments on this class.

Let us remind that the syntactic contradiction, extended to ${\mathrm{QC}}_{AN}$, is given by AND(ρ,NOT(ρ)), where p(AND(ρ,NOT(ρ)))=p(ρ)(1−p(ρ)). Following this idea, we want to characterize the elements $\rho_{\langle m,k\rangle }$ in $D_{Hol}$ such that $p\left(\rho_{m}\right)=1-p\left(\rho_{k}\right)$. In this way, if $\rho_{\langle m,k\rangle }$ is of the form $\rho_{\langle m,k\rangle }=\rho_{m}⊗\mathrm{NOT}\left(\rho_{m}\right),$ then ${\mathrm{AND}}_{Hol}\left(\rho_{\langle m,k\rangle }\right)=\mathrm{AND}\left(\rho_{m}⊗\mathrm{NOT}\left(\rho_{m}\right)\right)$. It generalizes the fuzzy extension of the syntactic contradiction in ${\mathrm{QC}}_{AN}^{Hol}$. We first introduce the following set

(25) $$D_{Hol}^{cont}=\left\{\rho_{\langle m,k\rangle }\in D_{Hol}:p\left(\rho_{m}\right)=1-p\left(\rho_{k}\right)\right\}.$$

The elements of $D_{Hol}^{cont}$ allow us to extend the notion of syntactic contradiction to ${\mathrm{QC}}_{AN}^{Hol}$ in the following way:

**Definition** **7.** *An expression of the form ${\mathrm{AND}}_{Hol}\left(\rho_{\langle m,k\rangle }\right)$ is said to be an holistic contradiction whenever $\rho_{\langle m,k\rangle }\in D_{Hol}^{cont}$*.

We note that an holistic contradiction can be characterized by a special value of $p({\mathrm{AND}}_{Hol}\left(\rho_{\langle m,k\rangle }\right))$ because

(26) $$\rho_{\langle m,k\rangle }\in D_{Hol}^{cont}\;iff\;p\left({\mathrm{AND}}_{Hol}\left(\rho_{\langle m,k\rangle }\right)\right)=\delta^{m,k}\left(\rho \right).$$

In fact, by Theorem 1, we have that:

$$\begin{array}{lll} p\left(\rho_{m}\right)=1-p\left(\rho_{k}\right) & iff & p\left({\mathrm{AND}}_{Hol}^{m,k}\left(\rho \right)\right)+\beta^{m,k}\left(\rho \right)=1-p\left({\mathrm{AND}}_{Hol}^{m,k}\left(\rho \right)\right)-\gamma^{m,k}\left(\rho \right)\\ & iff & p({\mathrm{AND}}_{Hol}^{m,k}\left(\rho \right)=1-p\left({\mathrm{AND}}_{Hol}^{m,k}\left(\rho \right)\right)-\gamma^{m,k}\left(\rho \right)-\beta^{m,k}\left(\rho \right)\\ & iff & p\left({\mathrm{AND}}_{Hol}^{m,k}\left(\rho \right)\right)=\delta^{m,k}\left(\rho \right). \end{array}$$

In other words, the notion of holistic contradiction is completely determined by the elements of $D_{Hol}^{cont}$. For this reason, if $\rho_{\langle m,k\rangle }\in D_{Hol}^{cont}$, $\rho_{\langle m,k\rangle }$ will be called as *holistically contradictory*.

A version of Theorem 1 for the elements of the set $D_{Hol}^{cont}$ is established below.

**Theorem** **2.** *Let $\rho_{\langle m,k\rangle }\in D_{Hol}^{cont}$. Then:*

*1*. *$p({\mathrm{AND}}_{Hol}\left(\rho_{\langle m,k\rangle }\right)=\frac{1-\beta^{m,k}\left(\rho \right)-\gamma^{m,k}\left(\rho \right)}{2}$,*
*2*. *$tr\left(T_{p}^{\left(m,k,1\right)}\left(\rho \right)\right)=\delta^{m,k}{\left(\rho \right)}^{2}-\beta^{m,k}\left(\rho \right)\gamma^{m,k}\left(\rho \right)$,*
*3*. *$0\leq p({\mathrm{AND}}_{Hol}\left(\rho_{\langle m,k\rangle }\right)\leq \frac{1}{2}$,*
*4*. *$p({\mathrm{AND}}_{Hol}\left(\rho_{\langle m,k\rangle }\right)=\frac{1}{2}$ iff $\beta^{m,k}\left(\rho \right)=\gamma^{m,k}\left(\rho \right)=0$ iff $p\left(\rho_{m}\right)=p\left(\rho_{k}\right)=\frac{1}{2}$ iff $tr\left(T_{p}^{\left(m,k,1\right)}\left(\rho \right)\right)=\frac{1}{4}$,*
*5*. *$p({\mathrm{AND}}_{Hol}\left(\rho_{\langle m,k\rangle }\right)=0$ iff $\beta^{m,k}\left(\rho \right)+\gamma^{m,k}\left(\rho \right)=1$ iff $tr\left(T_{p}^{\left(m,k,1\right)}\left(\rho \right)\right)=1-\beta^{m,k}\left(\rho \right)\left(1-\beta^{m,k}\left(\rho \right)\right)=1-\gamma^{m,k}\left(\rho \right)\left(1-\gamma^{m,k}\left(\rho \right)\right)$.*

**Proof.**  (1) Since $p({\mathrm{AND}}_{Hol}\left(\rho_{\langle m,k\rangle }\right)=\delta^{m,k}\left(\rho \right)$, by Theorem 1-1, $1=p({\mathrm{AND}}_{Hol}\left(\rho_{\langle m,k\rangle }\right)+\beta^{m,k}\left(\rho \right)+\gamma^{m,k}\left(\rho \right)+\delta^{m,k}\left(\rho \right)=2\delta^{m,k}\left(\rho \right)+\beta^{m,k}\left(\rho \right)+\gamma^{m,k}\left(\rho \right)$. Thus, $p({\mathrm{AND}}_{Hol}\left(\rho_{\langle m,k\rangle }\right)=\frac{1-\beta^{m,k}\left(\rho \right)-\gamma^{m,k}\left(\rho \right)}{2}$.

(2) Immediate from Theorem 1-4 and Theorem 2.

(3) Since $0\leq \beta^{m,k}\left(\rho \right)+\gamma^{m,k}\left(\rho \right)\leq 1$, by item 1, $0\leq p({\mathrm{AND}}_{Hol}\left(\rho_{\langle m,k\rangle }\right)\leq \frac{1}{2}$.

(4) By item 1, $p({\mathrm{AND}}_{Hol}\left(\rho_{\langle m,k\rangle }\right)=\frac{1}{2}$ iff $\beta^{m,k}\left(\rho \right)=\gamma^{m,k}\left(\rho \right)=0$ iff $p\left(\rho_{m}\right)=\delta^{m,k}\left(\rho \right)=1-p\left(\rho_{k}\right)=1-\delta^{m,k}\left(\rho \right)=\frac{1}{2}$ iff $tr\left(T_{p}^{\left(m,k,1\right)}\left(\rho \right)\right)={\frac{1}{2}}^{2}-0.$

(5) Immediate from item 1, item 2 and Theorem 2. □

The above theorem allows us to describe in a simple way the truth-functional behaviour of the holistic conjunction. It turns out to be very useful in the next section.

## 8. Werner States and Syntactic Contradiction

Werner states provide an interesting example of syntactic contradiction when a bipartition is considered. Werner states, firstly presented in [32] for two particles to discriminate between classical correlation and the Bell inequality satisfaction, have many interests for their applications in quantum information theory. Instances of this are entanglement teleportation via Werner states [33], the investigation on deterministic purification [34], etc.

**Definition** **8.** *Let H⊗H be a Hilbert space where dim(H)=n. A Werner state ρ defined on the space H⊗H is a density operator ρ such that, for any n-dimensional unitary operator U,*

$$\rho =\left(U⊗U\right)\rho \left(U^{†}⊗U^{†}\right).$$

Let us notice that any Werner states can be also written in terms of the identity and SWAP operators ([35] *§* 6.4.3) as follows:

(27) $$\rho =\rho_{w}^{\left(n^{2}\right)}=\frac{n+1-2w}{n(n^{2}-1)}I^{\left(n^{2}\right)}-\frac{n+1-2wn}{n(n^{2}-1)}{\mathrm{SWAP}}^{\left(n^{2}\right)},$$

where w∈[0,1] and ${\mathrm{SWAP}}^{\left(n^{2}\right)}=\sum_{i,j}|\psi_{i}\rangle \langle \psi_{j}|⊗|\psi_{j}\rangle \langle \psi_{i}|$, $|\psi_{i}\rangle$, $|\psi_{j}\rangle$ being vectors of the standard *n*-dimensional basis.

Let us consider the Werner state $\rho_{w}^{\left(2^{2n}\right)}$ in ${⊗}^{n+n}C^{2}$. Then, we can prove that (for more technical details, see ([29] Proposition 5.3).):

1. $p\left({\mathrm{AND}}_{Hol}\left({\rho_{w}^{\left(2^{2n}\right)}}_{\langle 2^{n},2^{n}\rangle }\right)\right)=\frac{2^{2n}+2^{n}\left(2w-1\right)-2}{4(2^{2n}-1)}$,
2. $p\left({\rho_{w}^{\left(2^{2n}\right)}}_{n}\right)=\frac{1}{2}$, where ${\rho_{w}^{\left(2^{2n}\right)}}_{n}$ is the partial trace of $\rho_{w}^{\left(2^{2n}\right)}$ with respect to the subspace ${⊗}^{n}C^{2}$,
3. $tr\left(T_{p}^{\left(2^{n},2^{n},1\right)}\left(M\left(\rho_{w}^{\left(2^{2n}\right)}\right)⊗P_{0}\right)\right)=\frac{w2^{n+1}-2^{n}-1}{4(2^{2n}-1)}$.

By item 2 and by Equation (25), it can be proved that the Werner state ${\rho_{w}^{\left(2^{2n}\right)}}_{\langle 2^{n},2^{n}\rangle }$ is a syntactic contradiction for each n∈N and for any w∈[0,1].

**Figure 1:**
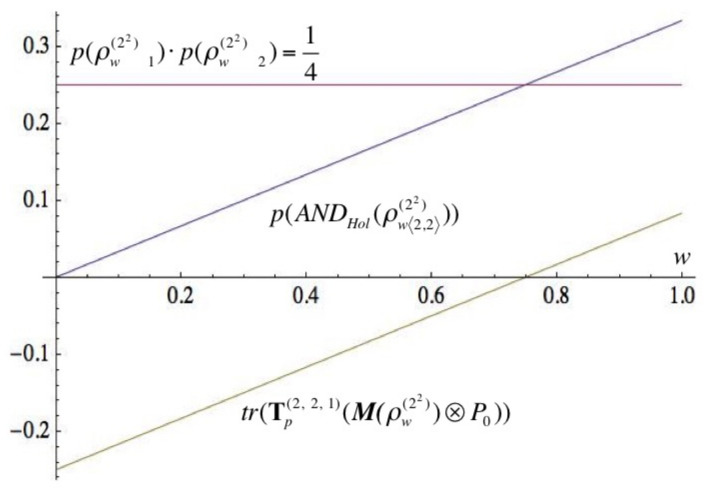
Werner as holistic contraddiction and incidence of $tr(T_{p}^{\left(2^{n},2^{n},1\right)}\left(M\left(\rho_{w}^{\left(2^{2n}\right)}\right)⊗P_{0}\right)).$

Figure 1 allows us to see the behavior of the Werner state $\rho_{w}^{\left(2^{2}\right)}$ as a syntactic contradiction taking into account the contribution of each parameter that defines the probability value $p({\mathrm{AND}}_{Hol}\left({\rho_{w}^{2^{2}}}_{\langle 2,2\rangle )}\right)$.

## 9. Conclusions

In this work, two semantical extensions of classical logic based on quantum computation with mixed states was investigated: the first, named ${\mathrm{QC}}_{AN}$, is a fuzzy type extension, while the second, named ${\mathrm{QC}}_{AN}^{Hol}$, is an improving of ${\mathrm{QC}}_{AN}$, where also holistic characteristics of bipartite quantum systems are considered. Both extensions are conceived from logical connectives for which natural interpretations are instances of Toffoli quantum gate acting on mixed states.

Formal aspects of these new logical systems were detailed in the paper, and they naturally suggest many interesting open questions and further developments in connection with different research areas. From the perspective of the philosophy of logic, ${\mathrm{QC}}_{AN}$ motivates new interpretations of fuzzy connectives in quantum computation. More precisely, some fuzzy logical systems, besides being related to the approximate reasoning or many-valued reasoning [36], also admit quantum probabilistic interpretations associated with quantum circuits. In the fuzzy context, notions like truth, tautology and logical consequences, may have another interpretation in the quantum computational framework. Technically speaking, ${\mathrm{QC}}_{AN}$ provides a good probabilistic description of circuits built on Toffoli quantum gates playing a similar role to classical logic in the digital techniques context. ${\mathrm{QC}}_{AN}$ deals with the ideal case where only factorizable states are taken into account. The holistic extension ${\mathrm{QC}}_{AN}^{Hol}$, instead, is able to describe combinational aspects of Toffoli quantum gate in a more general realistic way. As we have seen in Section 5, ${\mathrm{QC}}_{AN}^{Hol}$ is strongly related to the fuzzy systems that defines ${\mathrm{QC}}_{AN}$. Furthermore, this logical system provides an interesting connection between some holistic features arising from non-factorizable bipartite states and standard fuzzy logic. From an epistemological point of view, ${\mathrm{QC}}_{AN}$ and ${\mathrm{QC}}_{AN}^{Hol}$ can be considered as probabilistic type logic defining new kinds of quantum logic.

From an implementative perspective, these logical extensions can be very useful in quantum computing since the fuzzy content of ${\mathrm{QC}}_{AN}$ and ${\mathrm{QC}}_{AN}^{Hol}$ could be specially applied in fuzzy control [37], allowing for modelling the so-called Pelc’s game [38] (a probabilistic variant of Ulam’s game). It also suggests further developments in the study of error-correcting codes in the framework of quantum computation.
